# Can Social Communication Skills for Children Diagnosed With Autism Spectrum Disorder Rehearsed Inside the Video Game Environment of Minecraft Generalize to the Real World?

**DOI:** 10.2196/14369

**Published:** 2020-05-12

**Authors:** Lee Cadieux, Mickey Keenan

**Affiliations:** 1 School of Arts and Humanities Ulster University Londonderry United Kingdom; 2 School of Psychology Ulster University Coleraine United Kingdom

**Keywords:** autism, behavior analysis, serious games, social skills, gamification, Lego, neurodiversity, Minecraft, virtual worlds, virtual reality

## Abstract

In this paper, we outline opportunities within the video game environment for building skills applicable to real-world issues faced by some children. The game Minecraft is extremely popular and of particular interest to children diagnosed with autism spectrum disorder. Although the game has been used by support communities to facilitate the social interaction of children and peer support for their parents, little has been done to examine how social skills developed within the game environment generalize to the real world. Social Craft aims to establish a framework in which key social communication skills would be rehearsed in-game with a view to facilitating their replication in a similarly contained real-world environment. Central to this approach is an understanding of the basic principles of behavior and the engagement of a sound methodology for the collection of data inside and outside the respective environments.

## Introduction

Minecraft [1] is a procedurally generated open-world video game that employs distinctive block-building mechanics and a pixelated esthetic. It provides a constructivist environment whose earth, flora, and fauna may be mined, felled, or culled for component parts of ore, wood, or meat, respectively. These raw materials, in turn, may be crafted into food, tools, weapons, furniture, and building supplies. Blocks that may start as earth, vegetation, or animals may be harvested and broken down into their component parts such as coal from a ground block, wood from trees, or wool and mutton from sheep. These materials, in turn, may be combined into entirely new pieces with distinct in-game properties. Coal may be used to fuel a fire, wood may be fashioned into building supplies, and mutton may be cooked and eaten by the player to restore health. Trees may be chopped down and sawn into logs, which may be used to make sticks, and when combined with cobblestone blocks, may be fashioned into rudimentary tools such as axes or pickaxes. Animals such as cows may be culled, yielding beef, which in turn may be cooked and eaten, and whose hide may be fashioned into armor. Items are either carried in a personal inventory or stored in containers, which in turn may be placed in a home location, where players may craft further items or sleep.

Players navigate this cubist world using a similarly constructed block-person avatar, and most usually view their world from a first-person camera orientation, which reinforces a sense of belonging and immersion. As players gain experience, the environment evolves through a kind of Mesolithic, Paleolithic, and Iron Age culture. Multiple modes of play are offered, including Creative Mode, a more traditional block-building environment where blocks of every type are available in an open inventory and the more competitive Survival Mode in which players need to eat and sleep and defend themselves against a plethora of hostile nonplayer characters in the form of creepers, zombies, and witches to name a few. Players who perish from natural or unnatural causes, respawn in a recent home location, but lose any inventory items they are carrying.

The open-ended narrative structure of Minecraft, especially when used in Creative Mode, facilitates constructive play of a type that resembles real-life block-building play along the lines of Lego or similar interlinking toy systems. These real-world block building toys are similarly popular among children diagnosed with autism spectrum disorder (ASD) [2] and have been shown to be effective tools for social communication learning activity for children with ASD [3]. Minecraft provides a robust, if not, rudimentary system of text communication, but more often than not players seem to prefer to communicate and collaborate through voice-based group chat by using Skype audio or similar apps for simultaneous communication while playing together on a server-based system [4].

Although many other computer games exhibit a seemingly similar constructivist architecture and open-world platform to Minecraft, from some of the earliest text-based MUDs, such as Zork [5] to rich media 3D video games such as Elder Scrolls V: Skyrim [6], it is the open-ended narrative structure of the game and the in-world capabilities of game content creation, which draw parallels to the block-building toys referred to above.

This concept of avatar presence and interactive content creation is not without precedence and was explored in an earlier paper by Cadieux [7], which looked at sandbox platforms and the process of sandboxing in software development. A sandbox*,* in games-level design terminology, refers to the build area within a game environment, and sandboxing is a term that comes from software development, which describes a process of testing computer software in a safe, protected environment [7]. Certain game environments facilitate group constructivist interactions such as these, where the avatars of players may be collectively present to collaborate and build additional game content from within the game environment itself. Minecraft is one such video game.

The popularity of Minecraft with children with ASD seems fairly universal and, as such, the video game has been used by educators and ASD support communities to facilitate downtime from overwhelming classroom situations and to facilitate the social interaction of children [8]:

With Minecraft, you can really just be yourself. The social interactions, the relationships, the communication – everything just boils down to you and your keyboard.

Stuart Duncan, creator of Autcraft

Autcraft is an organization that hosts a Minecraft server, specifically geared toward users with ASD. The environment is managed through a system that employs administrators who patrol the environment from within the game using avatars with enhanced privileges. They monitor and police the Autcraft environment with a remit modeled on game-play etiquette and internet safety and provide a safe environment which entirely comprises children with autism, their parents, and family members. The Autcraft community limits its remit to policing its environment to reduce greifing or virtual antisocial behavior of its members and provides in-world support to the participants.

Within the safe Autcraft environment, individuals with autism can engage in behaviors normally associated with autism in the real world; there is no perceived need for anyone to do otherwise. Examples of behaviors associated with a diagnosis of autism include deficits in social-emotional reciprocity, deficits in nonverbal communicative behaviors in social interactions, and deficits in developing, maintaining, and understanding relationships [9]. Moreover, there are no clear evidence-based examples to date of Autcraft being used to facilitate social skills learning with children diagnosed with ASD beyond those related specifically to game-play behavior. Typically, children diagnosed with autism are given access to the gaming environment, and anecdotal reports are used to draw conclusions about how the gaming experience influences behavior in the real world. Often, these reports are positive [8], but it is difficult to determine if it was time spent in-game per se or if it was something within the architecture of the gaming experience itself that produced the outcome. There are many games that require social interaction to progress through different levels. However, these interactions may not target or emulate real-world social communication skills. More often than not, the skills acquired are specifically game-related skills or skills related to social interaction and are related to Web-based behavior or social networking; therefore, they would not necessarily generalize to real-world behaviors.

This begs the question as to whether it is at all possible for any skills acquired during emersion in a virtual world to generalize to the real world. Therapeutic applications of virtual reality (VR) show some promise in a variety of clinical populations. Examples include the treatment of phobias [10], restoring lost skilled movement in children after traumas and diseases [11,12], teaching social skills to adolescents with ASD [13], and safety skills [14]. Another huge area of research involves a related nonimmersive virtual world, one that involves basic principles of learning associated with watching others learn, observational learning [15]. Within this field, video modeling has proven highly effective in teaching children with autism to engage in behaviors observed in videos [16-19]. It can be argued that Autcraft designers could take advantage of the possibilities within the game environment for members to enhance their social communication skills or explore aspects of social relationships, such as making friends, reciprocity, and social engagement. This would be an important development at many levels. For example, a recent survey in the United Kingdom by the National Autistic Society (2016) showed that although many adults diagnosed with autism would like to work (77%), only 16% are in full-time paid work. A major contributing factor involved challenges related to social communication, including social difficulties presented by job interviews. At the very least, issues related to social interaction and communication norms in virtual settings present an empirical challenge filled with opportunities to address a range of research questions.

To set the scene for a discussion of what could be done, consider how the technological goals within a game are already mirrored in the real world. This is precisely the remit of the natural science of behavior analysis when it develops behavioral technology to facilitate social exchanges, where *technology* is understood as *the use and knowledge of tools, techniques, systems, or methods in order to solve a problem or serve some purpose* [20]. Currently, most evidence-based interventions for autism are based on the principles of applied behavior analysis (ABA) [21]. Politis et al [22] explain how the strategies that are integral to the design and implementation of an intervention program using ABA also form the basis of serious game design. They include defining and measuring behavior, recording and analyzing behavior, presenting corrective feedback, and dynamically adapting to student performance. However, whereas measurable outcomes are important for technological reasons in both gaming and real-world experiences, there is a major difference between the technologies employed in both. In the general game world, the algorithms do not record data with a view to showing the development of behavior as it moves toward a specified goal. Rather, the technology provides a simple set of rules for generating images on a computer screen. Social behavior either happens or it does not. In other words, algorithms are not designed to explicitly shape behavior toward a particular goal. In contrast, measurable outcomes that are monitored in the real world when using behavioral technology are essential for evaluating the viability of an educational program. These data provide a record of progress toward a specified goal. If a goal is not attained at any point in time, then the program may be adjusted in accordance with the data obtained, so as to increase the likelihood that the skills change in the direction of the goal.

Within a virtual world, the tools used in social communication (eg, using text and graphic symbols) are not those normally used in real-world contexts. That said, from a behavioral perspective, skills that could be enhanced within the virtual world should be viewed as classes of behavior rather than discrete behaviors. In other words, it would be a mistake to consider *behavior* as something distinct from *communication*. This is because the term *communication* comprises a class of behaviors, and as such, it is defined by consequences [23], not by the topographies of individual components. The empirical question, then, is whether classes of behavior that are established in a virtual world will generalize to the real world. Empirical questions such as this are not peculiar to VR or the use of avatars. In real-world behavioral interventions for children diagnosed with ASD, every effort is made to plan for generalization outside of the learning environment [24]. This is because skills acquired in one context do not necessarily generalize to new contexts [25].

Behavioral instructors state goals in terms of student behavior. Objectives such as *understanding* are translated into observable actions that indicate when the inferred *understanding* has been achieved. These *behavioral objectives* define skills that students should have mastered by their final evaluation. Next, shaping steps, that is, the actions students take to achieve competence, are analyzed. As students respond, instructions adjust to suit individual progress. [26]

This perspective on technology was pioneered by BF Skinner, who gave birth to the field of programmed instruction [27,28]. Software engineers who developed Minecraft programmed the digital technology to respond to behaviors from a person at a workstation. There is no specified goal to reach, as in programmed instruction. However, every session terminates with the goals of various kinds being accomplished in various ways. In many respects, this open-ended environment is part of the attraction for players, and it is ideal for identifying learning opportunities for skill development.

Some of the issues being addressed here might best be discussed in the context of the following hypothetical experiment. A number of young people diagnosed with ASD are given the opportunity to play Minecraft for a fixed period of time at school. Following this, they are given the opportunity to engage with each other in a free-play situation with a block-building toy, such as Lego. The research question would be whether the social behaviors that occurred in the game world also appear in the real world. In addition, it would be important to know their similarities and differences. Without data recording in the game world, there would be no way to address this question. If there were differences in behavior across both environments, then consider the possibility that some of the differences arose because behaviors associated with ASD came to the fore in the real world. If this was indeed the finding, then the conclusion would be that although the children like to play Minecraft, there are wasted opportunities within the game for addressing (ie, practicing) social skills. Addressing social skills deficits by developing game play that has different levels of competence needed for progression might increase the likelihood that these skills would generalize to the real world.

## Social Craft

Social Craft would seek to establish a framework in which key real-world social communication skills could be rehearsed in-game, with a view to facilitating their replication in a similarly contained real-world environment. Central to this approach is an understanding of how basic principles of behavior are already employed in most gaming experiences and other educational software, along with a robust methodology for the collection of data [29,30]. For example, from a digital programming point of view, there are software protocols for controlling the situations that demand executions of particular kinds of behavior and executions of particular kinds of responses to these behaviors. It goes without saying that it is because all of these behaviors and their associated consequences are monitored by software instructions that the *natural* to and fro of the gaming experience is ensured.

If researchers or therapists were to inhabit the game world by controlling a Minecraft avatar with enhanced administrator capabilities, they could witness first-hand behaviors exhibited by players as they interact. They would be able to assess behavior as they would in a real-world clinical or educational situation. Game world behaviors could be ameliorated in-game, just as real-world behaviors could be ameliorated in a clinical or educational situation. Game world data could be collected in-game, just as real world data may be collected in any social learning environment. The information collected might then be used to design an automated monitoring system that could detect examples of the kinds of difficulties encountered by some children within the game world with the goal of developing automated protocols that mimic the intervention provided by the avatar of the researcher or educationalist, offering a gamification-based solution. Alternatively, a set of protocols could be developed into a framework for volunteers for community or admin avatars to encourage behaviors related to positive social-communicative interaction. Basic principles of behavior could then be harnessed to shape these skills [31]. The kinds of social communication that would take place in the game environment are the same ones that would be reinforced in a real-world environment, for example, greeting, commenting, turn-taking, and cooperative play. What distinguishes the Minecraft environment from similarly structured environments is its open-ended structure that facilitates a type of play similar to those that may be engaged in within real-world environments. This cross-compatibility between the virtual world and the real world harbors the potential for the generalization of behavior between the real and virtual environments. Of course, there will be detractors who would argue that such an approach would destroy the game experience, but this misses the point. The gaming experience itself is an environment where contingencies of reinforcement operate, and it is the way these contingencies are designed that determine whether a game is enjoyable or not.

This analysis is not peculiar to Minecraft. Most games operate a points-based system, where various criteria must be reached before new reinforcers are made available. Interestingly, the principles that operate within games have been shown to be important for addressing many real-world social problems [32-34]. Commenting on the relation between the task of designing contingencies in real-world applications and in the game world, Morford et al [35] noted that the concept of gamification has not gone unnoticed.

For example, Skinner [36] commented on how video games are excellent examples of contingency programming, in which players interact with an arrangement of contingencies where their behavior is guaranteed to be reinforced, contacting salient and immediate consequences⸺players are almost guaranteed to be successful when they play a video game. Skinner mentioned how other aspects in our lives could be similarly designed, stating as follows [36]:

No one really cares whether Pac-Man gobbles up all those little spots on the screen…What is reinforcing is successful play, and in a well-designed instructional program students gobble up their assignments.

More recently, McGonigal [37] likewise recognized how we might capitalize on the success of games to address significant societal issues, stating as follows [37]:

If we take everything game developers have learned about optimizing human experience…I foresee games that fix our educational system. I foresee games that treat depression, obesity, anxiety, and attention deficit disorder…I foresee games that tackle global-scale problems such as climate change and poverty.

Intriguingly, the notion that there are design issues regarding controlling contingencies has a mixed reception depending on whether these contingencies are designed to operate in the game world or in the real world. The history of heated debate regarding real-world applications has usually centered on the politics of who should be in control (eg, Sidman [38], Skinner [39], and Wheeler [40]). In discussions on interventions for autism, the neurodiversity movement is particularly antagonistic toward behavior analysis [41], suggesting that contingencies of reinforcement can or should not be used to change behavior. Keenan [42], however, has a different perspective on the nature of behavior analysis. The argument is made that changing the behavior of a person diagnosed with autism is tantamount to not accepting them for who they are as a person. This argument can be made with any intervention, clinical or educational, for any person. If taken to the extreme, it promotes censorship of all practices that aim to empower people with skills that are useful to them, even parenting [43]. If the argument found its way into the gaming environment, it would stifle game development because it is not possible to design a game without intentionally controlling what a person does (ie, designing contingencies) within a game. And yet, by utilizing an app already embraced by the ASD community [8] and one which is used in schools and by ASD community support groups [44], the very use of Minecraft as a tool for supporting the reinforcement of a positive social communication skillset is in a way, an approach which is aligned to, rather than opposed to, particular aspects of neurodiversity. Social Craft may, then, offer the potential to bridge in some small way divergencies in rationale between the neurodiversity community and those who would seek to ameliorate issues related to social communication through a virtual form of behavioral intervention using the Minecraft platform.

Politis et al [22] drew attention to an additional way of offsetting difficulties when designing software for individuals diagnosed with ASD. They discuss the value of involving people with disabilities in the design process, incorporating what is called user-centered design (UCD). UCD, they explain, provides a way of furnishing designers with a better understanding of the end user. Within this framework, *technology developers and users meet as equals to promote reciprocal relationships, mutual learning, and empowerment of user* [22]. What better way to demonstrate your acceptance of a person than by tuning into any difficulties they may have when progressing through a game and augmenting their skills through the design of supportive contingencies. In other words, affording a player the opportunity to develop her abilities, or in game terms, to level up to better defend against enemies. This vision has parallels with living in the real world. Deficits in social skills are only deficits if they impede progress for the individual concerned, and only then should supportive contingencies be tailored to meet their needs, with their consent of course, or the consent of their primary carer, if a disability prevents them from communicating. This is precisely the approach of behavior analysis for gaming and living in the real world [45].

An alternative or additional way in which the Social Craft method could be modeled would involve the use of the Minecraft video game environment itself to host the interaction between the behavioral programmer and the player. In many ways, this could provide a more compatible environment in which to study the social interaction of an individual among their peer Minecraft players. Just as the players of the game act as operators of their keyboard or console in the physical world, and through these same interfaces control the actions and interaction activities of their avatars, therapists and researchers could operate through their keyboard or console in the real world and through their interfaces control avatars, with enhanced administrative capabilities in the same virtual environment.

In the real world, the therapist would base the intervention on an assessment of the candidate’s needs and social communication profile. From this information, a more detailed assessment would be conducted on the individual’s level of communication to help identify areas that have the potential for redress. Certain communication skills, such as facial expression, gestures, or tone of voice, do not have parallels in the video games environment of Minecraft, but others, such as eye contact, turn taking, and collaborative play, have parallel interactions in both the virtual and the physical environment. It is these which, when identified may be incorporated into a plan for intervention.

When applying the Social Craft methodology, the therapist identifies troublesome behaviors and uses positive reinforcement through a token system [46,47] to reinforce alternative behavior outcomes. Of course, some of these behaviors will be related to the game environment itself, but others hold the potential to generalize beyond the virtual play area. This is why the multiple workstation laboratory environment is very important to the Social Craft method. When therapist(s) and players are present together, not only in the computer suite but also in the same server installation of Minecraft, the participants are more likely to *feel a connection* between their avatars in the game and their presence in the laboratory with their peers and therapists. In behavioral terms, the more similar the environments, the more likely it is that generalization of skills will appear [48,49].

Virtual tokens could be modeled in the virtual world through modified component blocks that could emulate props from the game such as a precious metal ingot, helmet, or gemstone, which would only be available to players who had achieved points awarded for positive communicative outcomes. These would sit in a players’ inventory and could be used in-game by the players’ avatars, just as in-game props are carried or worn by Minecraft players. These in-game props would be empowered with real-world properties to allow the holders to extend game-play time, access new regions, or open higher-level inventories. Holders need to maintain positive game-play practice and maintain social-communicative interaction in order to retain the items.

A similar system could be employed in the real-world environment of the computer suite by using a parallel system of tokens and rewards. Real-world tokens could consist of life-sized Minecraft props or toys of similar design and color to those offered in the virtual environment. These could be used as badges and would empower the owner with similar real-world capabilities to those offered the players in the virtual environment by affording their holders extended game-play time or access premiums as long as the players maintained positive play practice and socially acceptable interaction within the real-world environment.

Virtual tokens already exist within the Minecraft environment as specific block types, such as life-sized prop toys of the same items. Off-the-shelf versions could be used or bespoke replicas manufactured. This system of virtual and real-world token and reward could provide a cross-platform method with which to shape and increase the likelihood of generalization of specific behaviors in both the virtual world and real-world environments.

Regarding data collection from any assessments that are made, in the gaming environment software automatically records aspects of performance to determine what consequences should or should not occur. In a sense, the algorithms at the heart of the software can be viewed as digitally analogous to an observer recording outcomes. This focus on measurable outcomes is central to strategies employed by behavior analysis when designing experiences in the real world. The following advice from a behavior analyst to a parent exemplifies the relevance of this point when it comes to explaining how to think about the measurement of a child’s social skills [50]:

Focus on observable outcomes. The fact that social skills need to be addressed is related to something observable. In other words, How do we know she needs improvement in (specific social skill)? There must be something observable that supports this objective. For instance, We know she needs improvement in engaging in social conversation with peers because she rarely if ever has such conversations. Taking this information and turning it into an observable outcome is essential. That is, “We will know when (specific social skill) has improved when we see that she (observable behaviors).”

Within the game world, sequences of interaction from the therapist player’s point of view may be recorded in real time and stored as video reference files to be analyzed in the real world by therapists or researchers. Specific behaviors that have been recorded in a game environment may be presented to and discussed with the player as examples of behaviors for potential amelioration.

For "higher functioning" children, the video-monitoring technique described by Lloyd et al [51] may be useful to assess a child’s metapragmatic knowledge of communicative intent. The video-monitoring technique involves a two-step procedure. The first step is to videotape a child in a communicative situation. Lloyd et al [51] situations such as a referential communication game or a peer-tutoring task, which involve child–child interactions, and adult–child interactions may also be explored. The second step is to watch the videotape with the child and probe the child's understanding of the speaker's effectiveness in expressing intent [52].

This in-game recording of player interaction would provide a gamified equivalent to the first step of videotaping a child in a communicative situation. Watching the in-game recording with the child would parallel the second step outlined in the video-monitoring technique described above by Wetherby-Prizant.

## Conclusions

In summary, within the Social Craft, the researcher or therapist would initially inhabit a Minecraft environment with enhanced Social Craft modifications and would use the enhanced capabilities of the environment to rehearse and reinforce social communication activities. At the same time, they would observe peer interaction and play, which could inform a set of protocols that could be followed by community or admin avatars or indeed could be gamified into an automated monitoring system. This game-world/real-world approach builds on platform game-world approaches used by other server-side ASD communities such as Autcraft but is geared toward developing social communication skills in-game and then reinforcing them in a parallel real-world environment. Social Craft seeks to establish mechanisms for collecting data in both the virtual world and the real world related to the generalization of social communication skills between worlds.

## Abbreviations

ABA: applied behavior analysis
ASD: autism spectrum disorder
UCD: user-centered design
VR: virtual reality
